# Healthcare Providers’ Perspectives on Patient–Healthcare Worker Communication in the Emergency Department: A Cross-Sectional Survey Study in Riyadh, Saudi Arabia

**DOI:** 10.7759/cureus.101434

**Published:** 2026-01-13

**Authors:** Abdulaziz Aburiyah, Ahmed Alsaeedi, Talal A Aljehaiman, Ghezlan Alotaibi, Reem Alshehri

**Affiliations:** 1 Emergency Medicine, King Abdulaziz Medical City Riyadh, Riyadh, SAU; 2 Nursing, King Abdulaziz Medical City Riyadh, Riyadh, SAU

**Keywords:** cultural competence, emergency department, healthcare communication, language barriers, nurse-physician differences, patient safety

## Abstract

Background: Effective communication between healthcare providers (HCPs) and patients is fundamental to quality emergency care. However, emergency department (ED) settings pose particular challenges due to time constraints, high patient volumes, and linguistic and cultural diversity. This study aimed to assess how physicians and nurses experience these communication barriers in a Saudi Arabian tertiary hospital ED and to identify factors associated with perceived communication difficulty. Methods: This cross-sectional survey was executed at King Abdulaziz Medical City, Riyadh, Saudi Arabia, from November to December 2024. A convenience sample of ED physicians and nurses was invited to complete a structured, self-administered questionnaire assessing communication barriers, empathy behaviors, and discharge practices. Data were analyzed using chi-square and Fisher’s exact tests for categorical variables, and Mann-Whitney U tests for ordinal variables. Statistical significance was set at p<0.05. Results: Among 157 respondents (78 physicians and 79 nurses), nurses reported greater communication difficulty (p<0.001) and higher perceived language and cultural barriers (both p<0.001). Interpreter access was lower among nurses (p<0.001). Although empathy behaviors were comparable, nurses reported higher participation in cross-cultural (51.9% vs. 10.3%) and empathy (48.1% vs. 28.2%) training. Language and cultural barriers demonstrated a moderate positive correlation (r=0.55, p<0.001), indicating a meaningful but not strong association. Furthermore, cumulative barrier exposure was associated with increased difficulty (p=0.008). Most providers (87.2%) delivered discharge instructions verbally. Conclusion: Communication experiences differed significantly between physicians and nurses, with nurses reporting greater difficulty and barrier exposure. These findings point out the need for targeted, evidence-based interventions such as improved interpreter access and communication training while recognizing that conclusions are limited by the study’s cross-sectional, single-center design.

## Introduction

Communication between healthcare providers (HCPs) and patients is a fundamental element of high-quality emergency service, with significant implications for patient health, patient safety, and patient satisfaction [1,2]. The healthcare environment, particularly the emergency department (ED), is regarded as challenging with high patient traffic, a short time frame, unpredictable workflow variability, and interruptions [2]. Within EDs, communication failures between clinicians have been shown to contribute to diagnostic delays, treatment inefficiencies, and reduced patient satisfaction, emphasizing the importance of effective team coordination. Language and cultural barriers are even greater challenges, particularly within a more diverse healthcare setting [3]. Patients with limited English proficiency receive care that is significantly different, including variations in the use of diagnostic tests, potential treatment delays, and different pain management [4, 5].

Language discordance has been shown to be more prevalent in emergency versus elective surgical cases [6]. However, interpreters’ availability and use continue to be variable among HCPs, resulting in communication gaps and leading to a negative impact, particularly for non-native speakers and patients with low health literacy [7, 8]. These challenges are often amplified in fast-paced environments such as EDs, where limited time and cultural or linguistic differences hinder accurate information exchange.

Physicians and nurses, despite sharing patient care responsibilities, face different challenges in communication and employ different practices [9]. That is, the physician has the essential responsibility for the disclosure of diagnosis and treatment plans, although nurses are sometimes the communicators who follow up on such disclosure, as described in [10]. Such differences in professional responsibilities are illustrated, thus clarifying the need for a communal approach in patient communication. The importance of communication training, cultural competency, and empathy is essential for effective healthcare provision, although important barriers remain in both the availability and effectiveness of such interventions [11, 12]. Interventions targeting communication have been associated with improvements in patient satisfaction and discussions on care decisions [13]. However, there is often minimal formal training for HCPs regarding cross-cultural communication despite the growing diversity of patient populations that they serve.

Communication at the time of discharge represents a fragile point in the ED patient care pathway, and research remains to identify gaps between guidelines and how these are being carried out in practice [14, 15]. The lack of complete discharge instructions is critical for the safety of the patient, and verbal instructions often omit important elements, such as precautions for return and medication adverse effects. The nature of instruction and evaluation of patient understanding can be quite different between providers, leading to preventable return visits and poor outcomes, especially when language barriers are present, and patients are handed discharge instructions in languages that they do not fully comprehend [16]. The overall impact of language barriers on HCPs and patient outcomes is poorly described, especially in areas like the Middle East, where linguistic and cultural barriers are particularly challenging. However, research in Middle Eastern emergency settings remains limited, and little is known about how HCPs themselves perceive and manage these communication barriers.

The purpose of this study was to assess perceived communication barriers and difficulties among HCPs in the ED of a tertiary academic hospital in Saudi Arabia. Specifically, the primary objective was to evaluate the perceived challenges and barriers to effective communication among ED physicians and nurses. The secondary objectives were to (1) examine the association between cumulative barrier exposure and reported communication difficulty and (2) explore the need for additional training in cross-cultural communication and empathy among ED staff. For the purposes of this study, communication difficulty was operationally defined as the self-reported degree of challenge experienced when interacting with patients, while barriers included language, cultural, and environmental factors limiting effective patient-provider communication.

## Materials and methods

Study design and setting

This cross-sectional study was performed in the ED of King Abdulaziz Medical City, a tertiary academic hospital located in Riyadh, Saudi Arabia. The cross-sectional survey design was appropriate for describing relationships and patterns, but does not permit causal inference between variables. The study sought to assess HCPs' views on patient communication in the ED, supplementing a prior study that explored the patient perspective on the same issue. Data were collected between November 2024 and December 2024.

Study population and sampling

The study population consisted of HCPs who had direct clinical contact with patients in the ED. The inclusion criteria comprised (1) physicians at any training level (interns, residents, or consultants) actively practicing in the ED during the study period and (2) registered nurses assigned to ED patient care duties. Exclusion criteria encompassed personnel not directly engaged in patient care, individuals occupying administrative or non-clinical positions, and those who opted out of participation. A convenience sampling method was used, inviting all eligible physicians and nurses working in the ED of King Abdulaziz Medical City to participate during the two-month data collection period. The target sample size of approximately 150 participants was determined based on the estimated number of eligible ED healthcare workers available during the study period and feasibility considerations related to staff schedules and anticipated participation rates. This approach ensured the broad representation of both the physician and nursing staff within the department. The survey was administered electronically in English using a secure online platform. Invitations were distributed to all eligible ED physicians and nurses through institutional email, with in-person reminders provided during staff meetings. Two follow-up email reminders were sent at one-week intervals to maximize participation. Out of approximately 180 eligible healthcare workers, 157 completed the survey, yielding an estimated response rate of 87%. English was selected as the survey language because it is the standard medium of professional communication in the study hospital, where all physicians and nurses are required to demonstrate English proficiency for clinical practice. Given this institutional language policy, translation was not deemed necessary, and language-related response bias was considered minimal. The study team reviewed staff demographic data and confirmed that English proficiency was consistent across physician and nursing groups. 

To maintain participant anonymity and encourage honest reporting, no identifying demographic or professional data (such as age, gender, or nationality) were collected. This approach was chosen to reduce social desirability bias and ensure participants felt comfortable sharing their communication experiences openly.

Data collection instrument

The research team developed a structured questionnaire after an extensive review of the literature on healthcare communication, empathy, and patient-provider interactions in emergency settings. The instrument was adapted and modified from previously published tools addressing similar communication constructs to ensure conceptual and content validity (Appendix A).

The questionnaire was given electronically in English and comprised three main sections. Section 1 included 16 core questions applicable to all respondents, addressing (1) perceived patient communication difficulties, (2) preferred communication methods with patients and companions, (3) time available for patient interaction, (4) frequency of interruptions, (5) language and cultural barriers, (6) accessibility of interpreter services, (7) the influence of patient characteristics and clinical setting, (8) the use of medical terminology, (9) the need for information repetition, (10) rapport-building and empathy behaviors, (11) beliefs regarding empathy and patient satisfaction, and (12) prior training in cross-cultural and empathy skills.

Section 2 consisted of 12 physician-specific items covering (1) self-introduction and professional disclosure, (2) communication of diagnosis, investigations, medications, potential side effects, and discharge instructions, (3) shared decision-making, (4) discharge instruction format, and (5) average duration of patient interviews. Section 3 comprised seven nurse-specific questions exploring (1) patient introductions, (2) explanation of treatments and disposition, (3) communication challenges about conditions and adherence, and (4) instances of miscommunication in pain management.

Items based on frequency used a four-point scale (1 = Never, 2 = Rarely, 3 = Often, 4 = Always/A lot), where higher scores indicated greater frequency or intensity of the measured behavior or barrier. The agreement items used a four-point Likert scale with the options ‘strongly disagree,’ ‘disagree,’ ‘agree,’ and ‘strongly agree.' Categorical items were used where appropriate. The draft instrument underwent expert review by three senior emergency physicians and two nursing faculty members to assess clarity, comprehensiveness, and relevance to the ED context. Based on their feedback, minor wording adjustments were made to improve readability and ensure face and content validity.

Prior to full data collection, a pilot test was conducted among 10 ED HCPs not included in the final study sample to evaluate comprehension, response consistency, and completion time. Internal consistency reliability for multi-item scales was assessed using Cronbach’s alpha, which demonstrated acceptable reliability (α = 0.82) across communication barrier and empathy subscales.

Ethical considerations

The Institutional Review Board of King Abdullah International Medical Research Center (reference: NRR24/026/10) reviewed and approved the study protocol. Before filling out the questionnaire, all participants gave their electronic consent. People could choose to take part, and they could leave at any time without any problems. The research team was the only one that could see the data, which were collected anonymously and stored safely.

Statistical analysis

Data were analyzed using IBM SPSS Statistics software (version 29.0; IBM Corp., Armonk, NY). Descriptive statistics were used to summarize study variables. Categorical variables were presented as frequencies and percentages, while continuous and ordinal variables were summarized as medians and interquartile ranges. Group comparisons between physicians and nurses were conducted using chi-square (χ²) or Fisher’s exact tests for categorical variables, and Mann-Whitney U tests for ordinal variables. Effect sizes (Cramer’s V for χ² tests and r for Mann-Whitney U tests) and 95% confidence intervals (CIs) were calculated to quantify the magnitude and precision of associations.

To minimize the risk of Type I error resulting from multiple comparisons, statistical significance was interpreted with caution, and findings were contextualized within the exploratory nature of the study. No formal correction was applied due to the descriptive design and limited sample size; however, primary outcomes (communication difficulty, perceived barriers, and access to interpreter services) were defined a priori. Potential confounders, including years of clinical experience, professional role, and native language, were considered in the interpretation of results but were not included in multivariable models, as the study was not powered for adjusted analyses. All tests were two-tailed, and a p-value < 0.05 was considered statistically significant.

## Results

Communication experiences among healthcare workers

A total of 157 healthcare workers participated in this study, comprising 78 physicians (49.7%) and 79 nurses (50.3%). Significant differences emerged between the two groups regarding perceived communication difficulties. Nurses reported substantially greater difficulty communicating with patients compared to physicians (16.5% vs. 1.3%, χ² = 15.7, p < 0.001, Cramer’s V = 0.38), representing a moderate-to-large effect size. Effect sizes and 95% CIs were calculated where applicable to provide a clearer interpretation of the magnitude of observed group differences. Physicians more frequently found it easier to communicate directly with patients (48.7% vs. 29.1%), whereas nurses were more likely to report equal ease with both patients and companions (49.4% vs. 35.9%, p=0.042). No significant differences were observed regarding perceived adequacy of time for communication, with approximately 60% of both groups reporting "somewhat" sufficient time (Table 1).

**Table 1:** Communication Experiences Among Healthcare Workers in the Emergency Department (N=157). This table compares communication experiences between physicians and nurses in the emergency department. Nurses reported significantly greater difficulty communicating and higher ratings for language and cultural barriers. Chi-square and Mann–Whitney U tests were used for group comparisons (p < 0.05). Data are presented as n (%) for categorical variables or median (IQR) for ordinal variables. P-values are derived from chi-square or Mann–Whitney U tests as appropriate. Frequency scale: 1 = Never, 2 = Rarely, 3 = Often, 4 = A lot; Agreement scale: 1 = Strongly disagree, 2 = Disagree, 3 = Agree, 4 = Strongly agree. † Items assessed on a frequency scale: 1 = Never, 2 = Rarely, 3 = Often, 4 = A lot; †† Items assessed on an agreement scale: 1 = Strongly disagree, 2 = Disagree, 3 = Agree, 4 = Strongly agree.

Variable	Overall (N=157)	Physician (N=78)	Nurse (N=79)	p-value
Difficulty communicating				<0.001
Not difficult	49 (31.2%)	35 (44.9%)	14 (17.7%)	
Somehow difficult	94 (59.9%)	42 (53.8%)	52 (65.8%)	
Very difficult	14 (8.9%)	1 (1.3%)	13 (16.5%)	
Easier to communicate with				0.042
Patient	61 (38.9%)	38 (48.7%)	23 (29.1%)	
Companion	29 (18.5%)	12 (15.4%)	17 (21.5%)	
Both	67 (42.7%)	28 (35.9%)	39 (49.4%)	
Time to communicate				0.633
Plenty	34 (21.7%)	16 (20.5%)	18 (22.8%)	
Somewhat	93 (59.2%)	49 (62.8%)	44 (55.7%)	
Not enough	30 (19.1%)	13 (16.7%)	17 (21.5%)	
Interrupted by others^††^	3.0 (3.0–4.0)	3.0 (3.0–3.75)	3.0 (3.0–3.5)	0.713
Language barrier^†^	2.0 (2.0–3.0)	2.0 (2.0–2.0)	3.0 (2.0–4.0)	<0.001
Cultural barriers^†^	2.0 (2.0–3.0)	2.0 (2.0–3.0)	3.0 (2.0–3.0)	<0.001
Access to interpreters^†^	2.0 (2.0–3.0)	2.0 (1.0–2.75)	3.0 (2.0–3.0)	<0.001
Patient characteristics difficulty^†^	3.0 (2.0–3.0)	3.0 (2.0–3.0)	3.0 (3.0–4.0)	0.103
Clinical setting affects communication^†^	3.0 (2.0–4.0)	3.0 (2.0–4.0)	3.0 (2.0–3.0)	0.051
Use medical terminology^†^	2.0 (2.0–3.0)	2.0 (2.0–3.0)	2.0 (2.0–3.0)	0.025
Patients ask to repeat the information^†^	3.0 (2.0–3.0)	3.0 (2.0–3.0)	3.0 (2.5–4.0)	0.001
Establish rapport^†^	3.0 (2.0–3.0)	3.0 (2.25–3.0)	3.0 (2.0–3.0)	0.106
Demonstrate empathy^†^	3.0 (3.0–4.0)	3.0 (3.0–4.0)	3.0 (3.0–4.0)	0.599
Empathy influences satisfaction^‡^	3.0 (3.0–4.0)	4.0 (3.0–4.0)	3.0 (3.0–4.0)	0.316
Cross-cultural training received	49 (31.2%)	8 (10.3%)	41 (51.9%)	<0.001
Empathy/communication training received	60 (38.2%)	22 (28.2%)	38 (48.1%)	0.016

**Figure 1:**
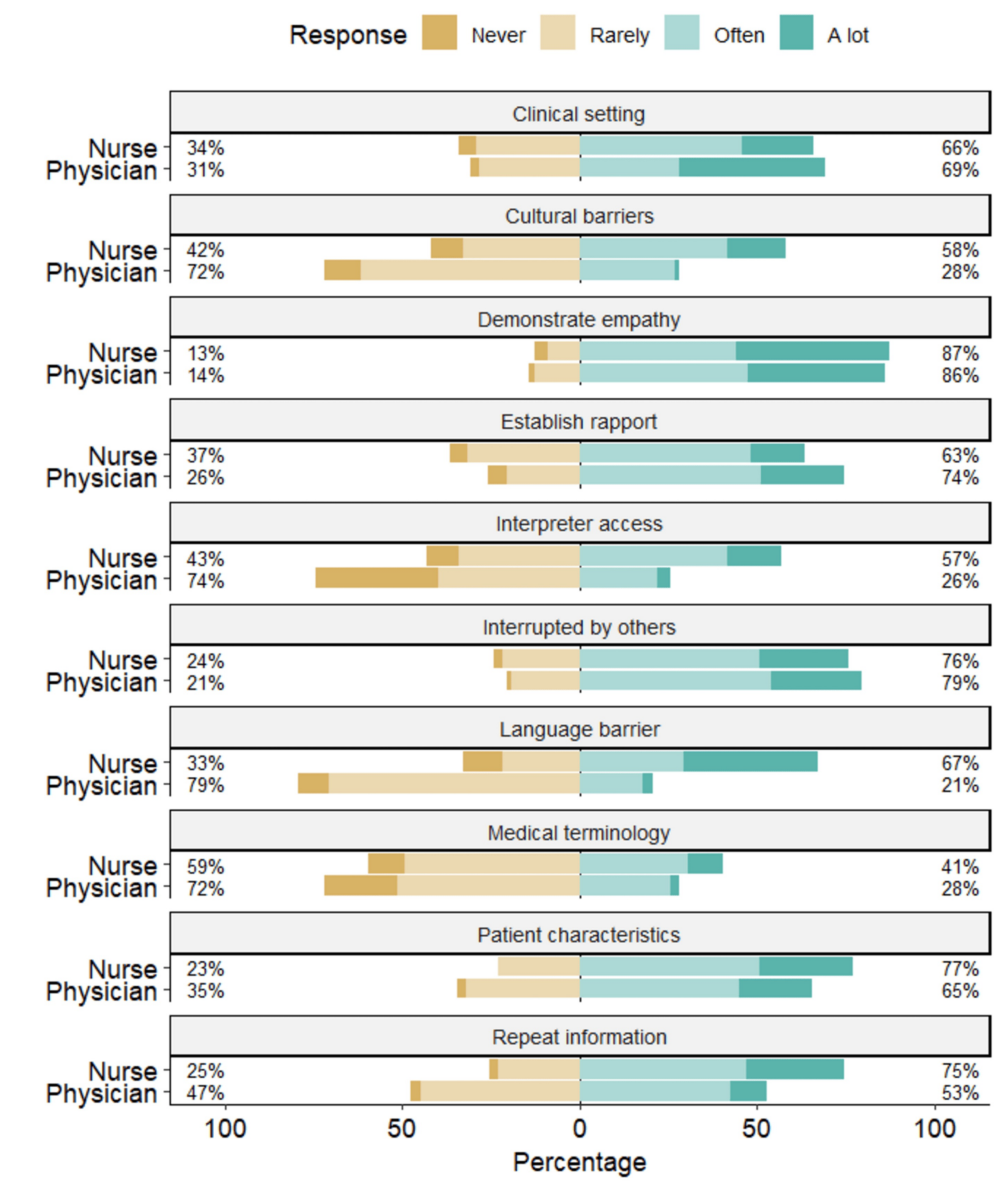
Communication Experiences Among Healthcare Workers in the Emergency Department (n = 157). This figure shows the distribution of physician and nurse responses to communication-related items. Nurses reported more frequent communication difficulties and greater exposure to language and cultural barriers compared with physicians. Bars represent percentages for each response category (Never, Rarely, Often, A lot). Group differences were analyzed using chi-square and Mann–Whitney U tests, with p < 0.05 considered statistically significant.

In Figure 1, percentages shown on the left represent the proportion of respondents in each role who selected "Never" or "Rarely." Percentages on the right represent those who selected "Often" or "A lot." Values are calculated separately for nurses and physicians for each communication item. Nurses perceived greater language barriers (median = 3.0 (Often) vs. 2.0 (Rarely), p < 0.001), cultural barriers (median = 3.0 (Often) vs. 2.0 (Rarely), p < 0.001), indicating more frequent barrier encounters, and limited interpreter access (median = 3.0 (Often) vs. 2.0 (Rarely), p < 0.001). Nurses also reported that patients more frequently requested information to be repeated (p=0.001). Despite these challenges, both groups demonstrated comparable levels of rapport-building and empathy, with no significant differences in self-reported empathetic behavior or beliefs about empathy's influence on patient satisfaction. Notably, nurses had substantially higher rates of formal training in cross-cultural communication (51.9% vs. 10.3%, p<0.001) and empathy/communication skills (48.1% vs. 28.2%, p=0.016).

Physician-only questions

**Figure 2:**
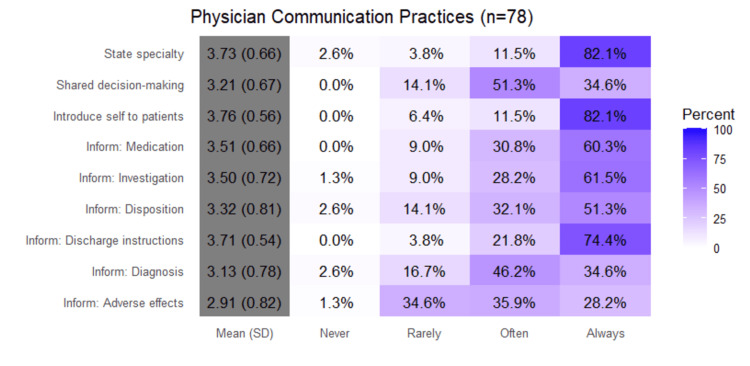
Physicians’ Communication Practices (n = 78) This figure illustrates the frequency with which physicians engaged in specific communication behaviors, including self-introduction, shared decision-making, and discussions of diagnoses, medications, and adverse effects. Responses were measured on a four-point scale (Never, Rarely, Often, Always). The results show high consistency across most items, with the greatest variability observed in providing discharge instructions and discussing adverse effects. Bars represent percentages, and statistical comparisons were descriptive.

In Figure 2, percentages represent the proportion of physicians selecting each response category (Never, Rarely, Often, Always). Higher mean scores indicate more frequent communication practices. Physicians frequently introduced themselves to patients (3.76 ± 0.56; 82.1% always, 11.5% often) and stated their specialty (3.73 ± 0.66; 82.1% always). Respondents were less consistent (3.21 ± 0.67; 51.3% often, 14.1% rarely) in stating their level of training. Communicating diagnoses showed moderate frequency (3.13 ± 0.78; 46.2% often, 34.6% always). Information about medications (3.51 ± 0.66; 60.3% always) and investigations (3.50 ± 0.72; 61.5% always) was routinely provided. Discussion of adverse effects was the least consistent practice (2.91 ± 0.82; 28.2% always, 35.9% often). Disposition communication was performed regularly (3.32 ± 0.81; 51.3% always), while discharge instructions were provided most consistently (3.71 ± 0.54; 74.4% always). Shared decision-making involvement showed moderate engagement (3.21 ± 0.67; 34.6% always, 51.3% often).

**Figure 3:**
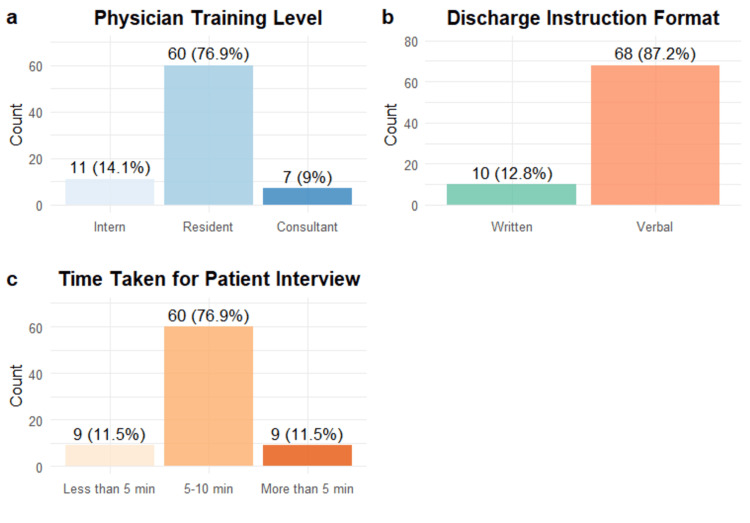
(a) Physician Training Level, (b) Discharge Instruction Format, and (c) Time Taken for Patient Interview (n = 78). This figure presents the distribution of physicians by training level, preferred discharge instruction format, and time spent conducting patient interviews. Most participants were residents (78.9%) and predominantly provided verbal discharge instructions (87.2%). The majority reported interviews lasting less than 10 minutes (76.3%). Bars represent frequencies, with percentages shown above each category. Data are descriptive.

In Figure 3, the majority of physicians were residents (76.9%, n = 60), followed by a smaller number of interns (14.1%, n = 11) and consultants (9.0%, n = 7). Discharge details were mainly given verbally (87.2%, n = 68), whereas written instructions were utilized significantly less frequently (12.8%, n = 10). Most interviews spanned 5-10 minutes (76.9%, n = 60), with equal percentages noting times of under five minutes (11.5%, n = 9) or exceeding 10 minutes (11.5%, n = 9).

Nurse-only questions

**Figure 4:**
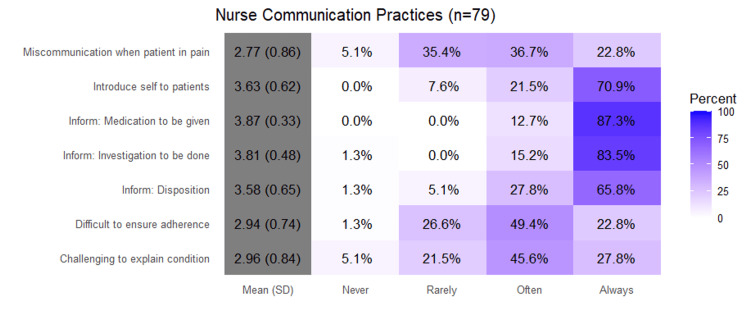
Nurses’ Communication Practices (n = 79). This figure presents nurse responses to survey items assessing communication behaviors, including self-introduction, explaining medications and investigations, and ensuring treatment adherence. Responses were rated on a four-point frequency scale (Never, Rarely, Often, Always). Most nurses reported consistently engaging in communication behaviors, particularly when discussing medications and patient disposition. However, challenges were noted in interactions with patients in pain or when adherence required reinforcement. Bars represent percentages.

In Figure 4, percentages represent the proportion of physicians selecting each response category (Never, Rarely, Often, Always). Higher mean scores indicate more frequent communication practices. Nurses introduced themselves to patients frequently (3.63 ± 0.62; 70.9% always, 21.5% often) and consistently informed patients about medications (3.87 ± 0.33; 87.3% always) and investigations to be done (3.81 ± 0.48; 83.5% always). Communicating disposition plans occurred regularly (3.58 ± 0.65; 65.8% always, 27.8% often). Miscommunication when patients were in pain was reported occasionally (2.77 ± 0.86; 36.7% often, 22.8% always). Ensuring adherence posed moderate difficulty (2.94 ± 0.74; 49.4% often, 22.8% always), and explaining conditions was sometimes challenging (2.96 ± 0.84; 45.6% often, 27.8% always).

As shown in Table 2, physicians and nurses showed similar patterns in introducing themselves to patients, with most reporting always doing so (76.4% overall; physicians 82.1%, nurses 70.9%, p=0.214). Clear differences emerged in informing patients about medications, where nurses demonstrated higher consistency, with 87.3% always providing this information compared with 60.3% of physicians (p<0.001). A similar pattern was observed for informing patients about investigations, with 83.5% of nurses versus 61.5% of physicians reporting always doing so (p=0.001). Communication regarding disposition showed no statistically significant group difference (p=0.124), although nurses again reported higher consistency (65.8% always) compared with physicians (51.3% always). Rare or never responses were minimal across all items, reflecting generally high communication engagement across both roles.

**Table 2:** Comparison of Overlapping Communication Practices Between Physicians and Nurses (N=157). Comparison of overlapping communication practices between physicians and nurses (N=157). Both groups demonstrated high consistency in key communication behaviors such as patient introductions and discharge discussions, although significant differences were observed in medication and investigation-related communication. Group comparisons used chi-square tests with p < 0.05 considered statistically significant.

Variable	Overall (N=157)	Physician (N=78)	Nurse (N=79)	p-value
Introduce self to patients				0.214
Rarely	11 (7.0%)	5 (6.4%)	6 (7.6%)	
Often	26 (16.6%)	9 (11.5%)	17 (21.5%)	
Always	120 (76.4%)	64 (82.1%)	56 (70.9%)	
Inform: Medication				<0.001
Rarely	7 (4.5%)	7 (9.0%)	0 (0.0%)	
Often	34 (21.7%)	24 (30.8%)	10 (12.7%)	
Always	116 (73.9%)	47 (60.3%)	69 (87.3%)	
Inform: Investigation				0.001
Never	2 (1.3%)	1 (1.3%)	1 (1.3%)	
Rarely	7 (4.5%)	7 (9.0%)	0 (0.0%)	
Often	34 (21.7%)	22 (28.2%)	12 (15.2%)	
Always	114 (72.6%)	48 (61.5%)	66 (83.5%)	
Inform: Disposition				0.124
Never	3 (1.9%)	2 (2.6%)	1 (1.3%)	
Rarely	15 (9.6%)	11 (14.1%)	4 (5.1%)	
Often	47 (29.9%)	25 (32.1%)	22 (27.8%)	
Always	92 (58.6%)	40 (51.3%)	52 (65.8%)	

In Table 3, language barriers and cultural barriers demonstrated the strongest association (r=0.550, p<0.001), indicating these challenges frequently co-occur. Language barriers were also significantly correlated with use of medical terminology (r=0.316, p<0.001) and patients asking to repeat information (r=0.320, p<0.001), suggesting a coherent pattern where language difficulties lead to comprehension challenges requiring clarification. Cultural barriers showed similar associations with medical terminology use (r=0.288, p<0.001) and information repetition (r=0.277, p<0.001). The difficulty related to patient characteristics correlated moderately with both interruptions (r=0.293, p<0.001) and cultural barriers (r=0.286, p<0.001). Establishing rapport and demonstrating empathy were significantly correlated (r=0.256, p<0.01), forming a distinct positive communication dimension. Demonstrating empathy was also associated with patients requesting information repetition (r=0.252, p<0.01).

**Table 3:** Correlation Matrix of Communication Items (N=157). This table displays Spearman correlation coefficients (ρ) for relationships among ten communication-related variables, including language barriers, cultural barriers, interpreter access, empathy, and rapport establishment. Positive values represent direct relationships, where increased difficulty in one domain corresponds to higher scores in another. Statistically significant values (p < 0.05) are indicated in bold. A moderate positive correlation was observed between language and cultural barriers (ρ = 0.550, p < 0.001), suggesting that these challenges frequently co-occur in clinical interactions. P-values indicate the level of statistical significance. Asterisks denote significance levels as follows: ***p < 0.001, **p < 0.01, *p < 0.05.

Variable	1	2	3	4	5	6	7	8	9	10
1. Interrupted by others	—									
2. Language barrier	0.112	—								
3. Cultural barriers	0.146	0.550***	—							
4. Interpreter access	−0.158*	0.121	0.039	—						
5. Patient characteristics	0.293***	0.160*	0.286***	0.018	—					
6. Clinical setting	0.173*	−0.089	0.102	−0.133	0.123	—				
7. Medical terminology	0.138	0.316***	0.288***	−0.095	0.168*	−0.007	—			
8. Repeat information	0.208**	0.320***	0.277***	0.055	0.167*	−0.027	0.188*	—		
9. Establish rapport	0.015	0.127	0.129	0.026	0.014	0.070	0.009	0.059	—	
10. Demonstrate empathy	0.123	0.183*	0.047	−0.004	−0.006	−0.008	−0.064	0.252**	0.256**	—

**Figure 5:**
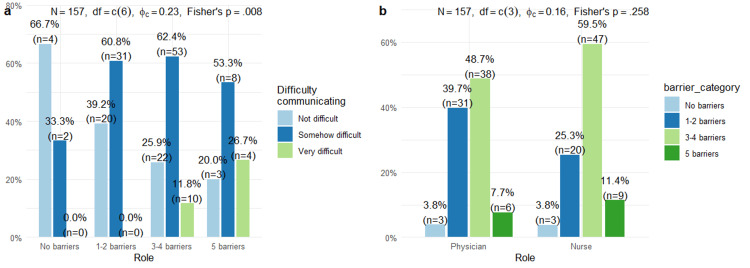
Relationship Between Communication Barriers and (a) Perceived Difficulty and (b) Professional Role (n = 157). This figure illustrates the relationship between the number of reported communication barriers and perceived communication difficulty, as well as differences by professional role. Panel (a) demonstrates a dose–response pattern, where increased exposure to language, cultural, and interpreter-related barriers corresponded to greater reported communication difficulty (Fisher’s p = 0.008, φc = 0.23). Panel (b) compares barrier frequency between physicians and nurses, showing that nurses experienced more cumulative barriers than physicians (Fisher’s p = 0.016). Bars represent percentages, and higher values indicate greater exposure or difficulty.

Figure 5 illustrates communication difficulty stratified by barrier count (Panel a) and the distribution of barriers by role (Panel b). Barrier categories were derived from frequency ratings ≥3 ("Often"/"A lot") on five items: language barrier, cultural barriers, low interpreter access, patient characteristics, and clinical setting. Fisher's exact test was used due to expected cell counts <5. φc = Cramer's V effect size. A significant association was observed between cumulative barrier exposure and perceived communication difficulty (Fisher's p=0.008, φc=0.23). Among respondents reporting no barriers, 66.7% rated communication as "not difficult," and none reported "very difficult." This pattern shifted progressively with increasing barrier counts: those with one to two barriers showed 39.2% "not difficult" with still no "very difficult" responses, while three to four barriers yielded 11.8% "very difficult" ratings. Healthcare workers experiencing all five barriers demonstrated the greatest difficulty, with 26.7% reporting "very difficult" and only 20.0% reporting "not difficult." Despite this clear dose-response pattern, barrier category distribution did not differ significantly by professional role (Panel b; Fisher's p=0.258), with both physicians and nurses most commonly reporting three to four barriers (48.7% and 59.5%, respectively).

## Discussion

This research showed a distinction in the experiences of communication challenges faced by physicians and nurses within a Saudi Arabian ED. The nurses experienced a significantly higher number of language and cultural barriers than the physicians. Additionally, the nurses reported limited availability of communication assistance, as patients were more often asking for a repeat of instructions. Despite such experiences, the level of empathy displayed by the two groups appeared similar. Nurses received significantly more cross-cultural communication and empathetic skills training than physicians. The dose-response relationship for accumulated barrier exposure, which increases on a rising scale, showed that individuals with greater exposure to barriers experienced significantly more challenges. These findings should be interpreted cautiously, as the observed differences may be influenced by the nature of professional roles, task expectations, and exposure time rather than true differences in communication ability.

The fact that the nurses reported challenges in communication is not entirely surprising, as they are the patients' point of contact with healthcare services, with the longest exposure at the patients' bedside, educating them on how to follow healthcare commands [9]. In the context of the ED, nurses are responsible for making life-or-death communications, particularly in high-stakes situations where language barriers exist in healthcare. Inconsistencies in the reported communication challenges may also result from differences in interaction modes; doctors typically have limited time during short diagnostic calls, whereas nurses spend significant time with patients in the healthcare setting, regularly facing a variety of challenges in their interactions [17]. An overwhelming amount of literature has attested that HCPs interpret challenges within healthcare communications differently depending on the healthcare professional setting/context [18].

Barriers of language and culture co-occurred extensively, with a high degree of association with the use of medical terminologies, as well as patients' requests for repeat statements. Such a grouping of barriers tends to indicate that difficulties with communication might work together, thereby exerting a combined, rather than a single, impact. Lack of proficiency in English has been suggested to be a factor contributing to inequities within a variety of domains of healthcare, such as the use of diagnostic procedures, treatment, and healthcare outcomes [4, 7]. The observation that language barriers were more pronounced for nurses, despite receiving more cross-cultural training, seems to suggest that while training is a necessary component, a lack of supportive infrastructure, such as availability, might serve as a hindrance. The addition of interpreter services within the electronic healthcare record system has been suggested to have the potential to increase language accessibility, resulting in increased use of interpreter services, with reduced waiting times [19]. However, ED nurses suggested the existence of environmental, temporal, and organizational barriers to accessing language services [20, 21].

Despite the surprising findings that nurses receive more training on formal communication, they perceive more difficulties, which is worth noting. This contradiction might be attributed to several reasons, such as a better appreciation of the deficiency following training and the relevance of nursing tasks to high-level communication, as well as organizational factors that might suppress the application of skills acquired. Research on incomplete care experienced by physicians reveals that when there is a sense of a constrained timeframe, especially regarding diagnoses and treatment options, supportive care, potentially involving communication, may be neglected [18]. Training programs, when combined with environmental changes and educational instruction, have improved care decisions [13].

An analysis of physician communication behavior revealed that self-disclosure, as well as disclosure of medication information, is generally high, but disclosure of training levels and potential contraindications is less consistent. The observation that patients mostly received verbal instructions, with only a small proportion receiving written instructions, is concerning, especially in light of the literature that shows a significant improvement in patient understanding and recall when instructions are conveyed verbally as well as in written form [14, 16]. Return precautions and patient comprehension are lacking worldwide, with many patients not receiving return precautions when discharged [15].

The relationship of dose-response to barrier exposure and communication difficulty has important implications for interventions. The highest perceived degree of communication difficulty is when HCPs experience all five barriers, implying interventions that aim at multiple barriers may be more effective than single ones. Effective interventions for improved communication might therefore require combining interventions with changes in the environment, structured interventions, and leader engagement to bring lasting behavior change [22, 23]. Training in empathy with fresh approaches such as virtual reality has been proven to result in improved physician empathy with underserved patients [24]. Moreover, personal perceptions of what constitutes compassionate care vary significantly with different specialty practices, which has implications that interventions in healthcare communication need to specifically address differing needs in different environments [25].

This study had a few limitations. The cross-sectional nature of the study means that inferences cannot be made about factors that led to barriers or impacted communication difficulties, and, therefore, the relationships identified should be interpreted as descriptive associations rather than causal effects. The use of self-reporting can be susceptible to social desirability bias, potentially leading to an underestimation of communication issues. The single-site nature of the tertiary academic center may limit generalizability to other healthcare settings in Saudi Arabia as well as internationally. The utilization of a convenience sample constrains the breadth of inference and may not accurately represent the entirety of healthcare personnel. Additionally, the absence of demographic and professional data (e.g., age, gender, years of experience, and nationality) limits the ability to assess whether communication experiences varied by these factors, and therefore, generalizability to other settings should be interpreted with caution. Additional limitations include the potential for non-response bias and the absence of formal psychometric validation for the study instrument. Furthermore, no adjustments for multiple comparisons were applied, and results should therefore be interpreted with appropriate caution. Future research should consider the use of longitudinal or interventional designs, as well as multi-site samples, to assess communication outcomes and examine factors within institutions that impact improved communication.

## Conclusions

In conclusion, what emerges from this research is that there are significant discrepancies in communication experiences that exist between ED physicians and nurses, with nurses being subjected to far more difficulties than physicians, who have a significantly greater rate of formal training. The existence of multiple layers of barriers in the process of patient communication is a compelling reason to support the need for holistic interventionist approaches that blend training with infrastructure support, such as a reliable interpreter service, in order to overcome such challenges. These conclusions should be interpreted within the context of this study’s design and setting, as they are based on self-reported data from a single tertiary center and may not be generalizable to other healthcare environments.

## Figures and Tables

**Figure 6:**
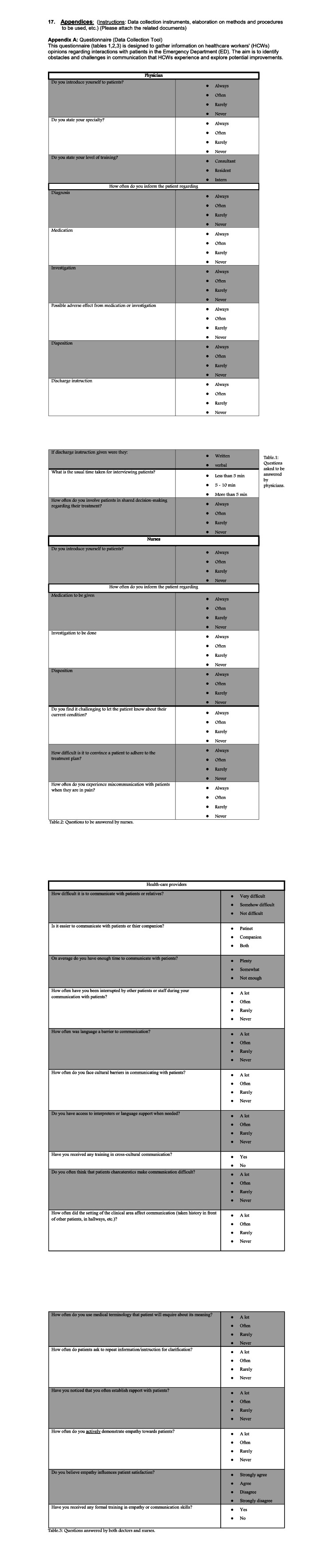
Study Questionnaire

## Appendices

Appendix A
